# Supplementary material for: Future flooding tolerant rice germplasm: Resilience afforded beyond Sub1A gene
Source: Plant Genome. 2025 May 8;18(2):e70040. doi: 10.1002/tpg2.70040 (PMC12060086; doi:10.1002/tpg2.70040)
Supplement: Supplementary file 1 — Figure S1: A schematic representation of the phenotypic screening of the whole collection of 6,274 genotypes. Figure S2: Diagram illustrating the stagnant flooding screening protocol adopted in this study Figure S3: Distribution of days to maturity and plant height (cm) among the 89 elite genotypes under flooding and normal conditions. Figure S4: Visual representation of the tolerant stagnant flooding genotypes grown in the IRRI‐HQ field during the 2023 DS [file TPG2-18-e70040-s003.docx]

**Supplementary Information**

**Future flooding tolerant rice germplasm: resilience afforded beyond *Sub1A* gene**

Mahender Anumalla^1,2^, Apurva Khanna^1^, Margaret Catolos^1^, Joie Ramos^1^, Ma Teresa Sta. Cruz^1^, Challa Venkateshwarlu^2^, Jaswanth Konijerla^2^, Sharat Kumar Pradhan^3^, Sushanta Kumar Dash^3^, Yater Das^4^, Dhiren Chowdhury^5^, Sanjay Kumar Chetia^6^, Janardan das^7^, Phuleswar Nath^7^, Girija Rani Merugumala^8^, Bidhan Roy^9,^ Navin Pradhan^10^, Monoranjan Jana^11^, Indrani Dana^11^, Suman Debnath^11^, Anirban Nath^12^, Suresh Prasad Singh^13,^ Khandakar Md Iftekharuddaula^14^, Sharmistha Ghosal^14^, Mohammad Ali^15^, Sakina Khanam^16^, Md Mizan Ul Islam^17^, Muhiuddin Faruquee^17^, Hosna Jannat Tonny^17^, Md Rokebul Hasan^18^, Anisar Rahman^14^, Jauhar Ali^1^, Pallavi Sinha^1,2^, Vikas Kumar Singh^1,12^_,_ Mohammad Rafiqul Islam^17^, Sankalp Bhosale^1^, Ajay Kohli^1^, Hans Bhardwaj^1^, and Waseem Hussain^1^*

**
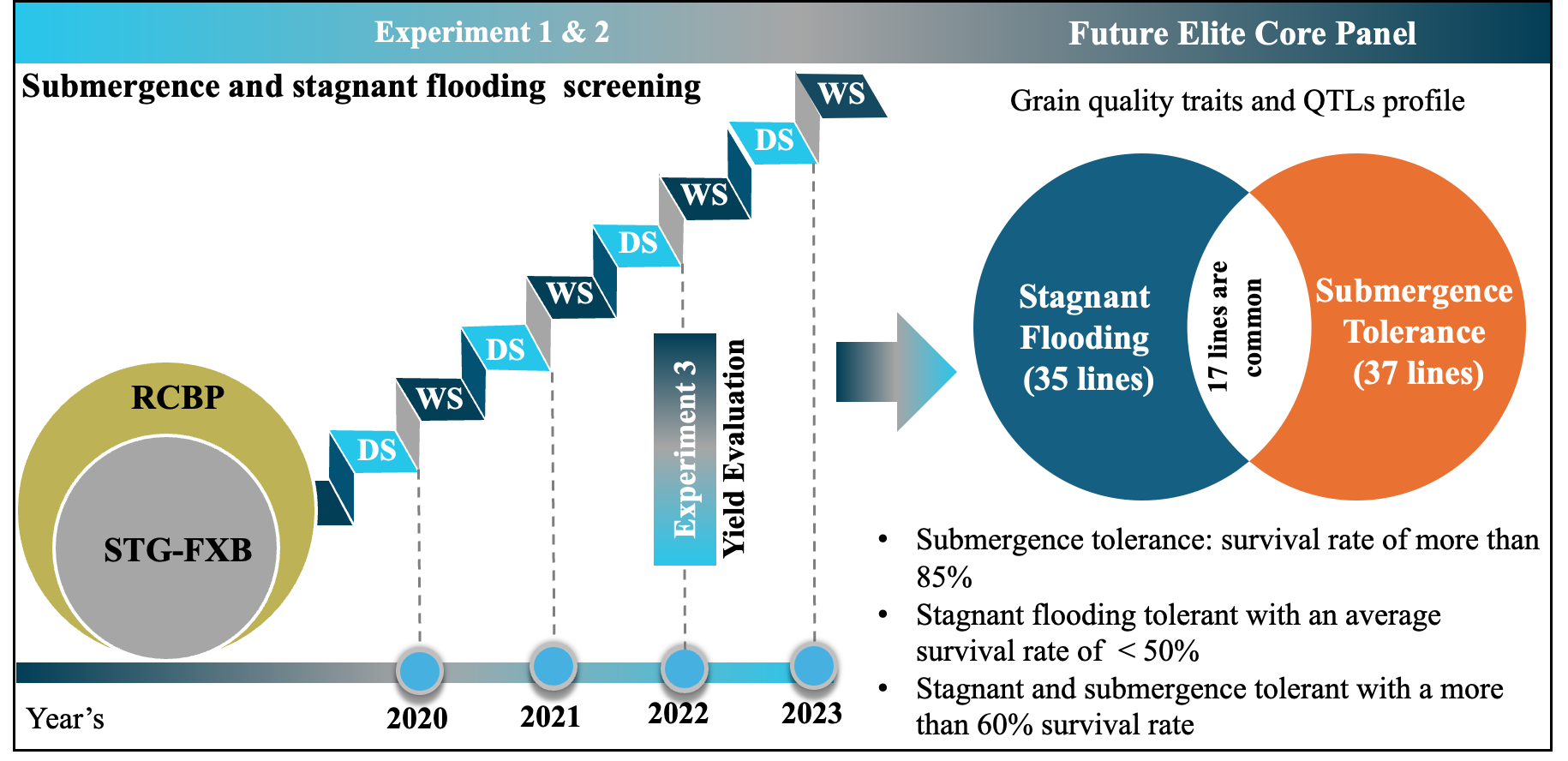
Figure S1.** A schematic representation of the phenotypic screening of 6,274 genotypes over the years and seasons at IRRI-HQ, Philippines. From 2020 to 2023, experiments were conducted at IRRI-HQ during the dry and wet seasons for submergence and stagnant flooding studies. In 2022 and 2023, yield experiments of the top identified elite genotypes were performed to evaluate the genotypes for yield, grain quality, and other important agronomic traits.


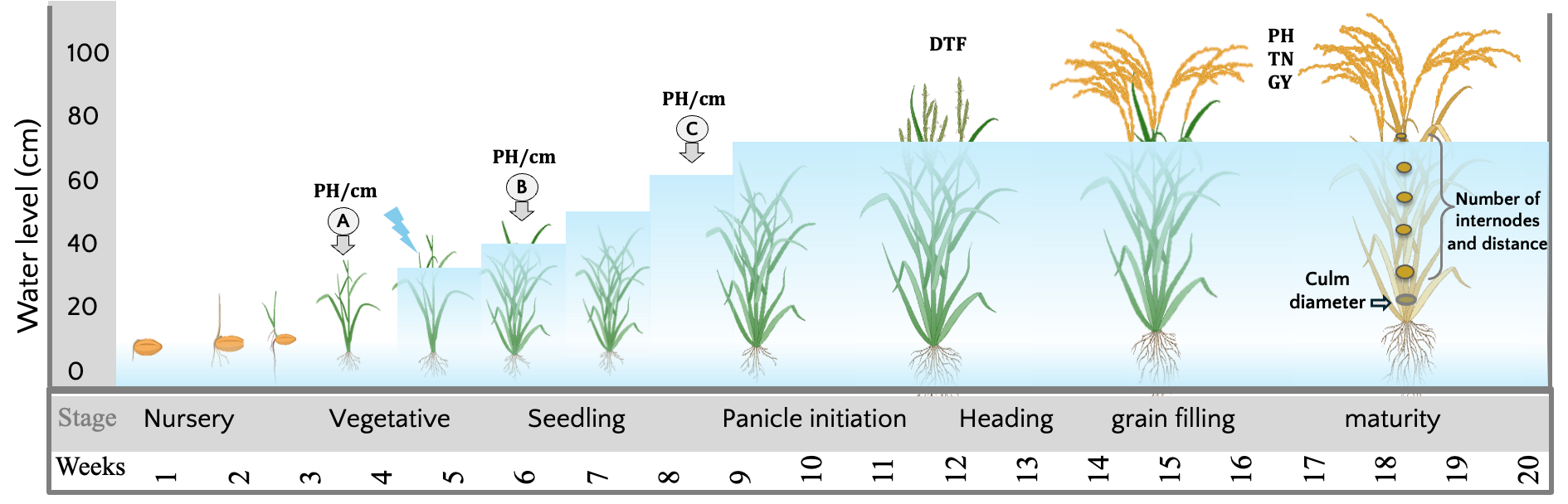


**Figure S2.** Diagram illustrating the stagnant flooding screening protocol used in this study. In this screening, 21-day-old seedlings are transplanted, and the water depth is increased weekly by 5 cm, reaching 40 cm by 56 days after transplanting (DAT), 50 cm by 63 DAT, and 70 cm by 70 DAT till maturity.


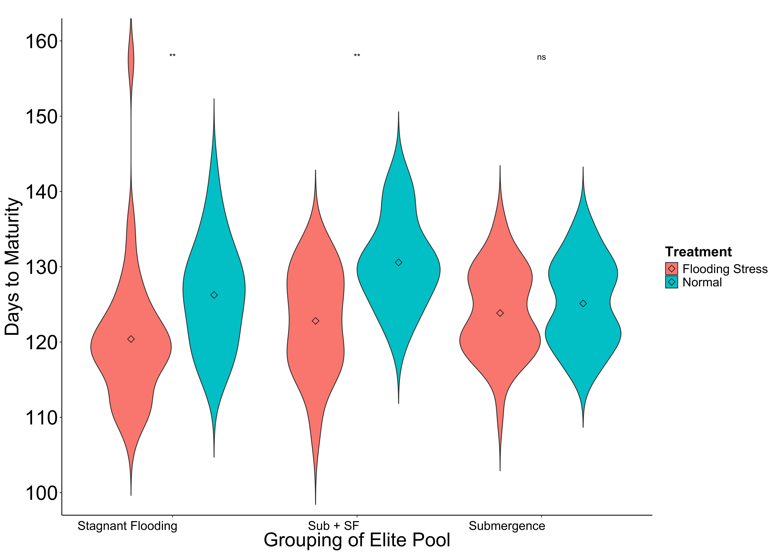

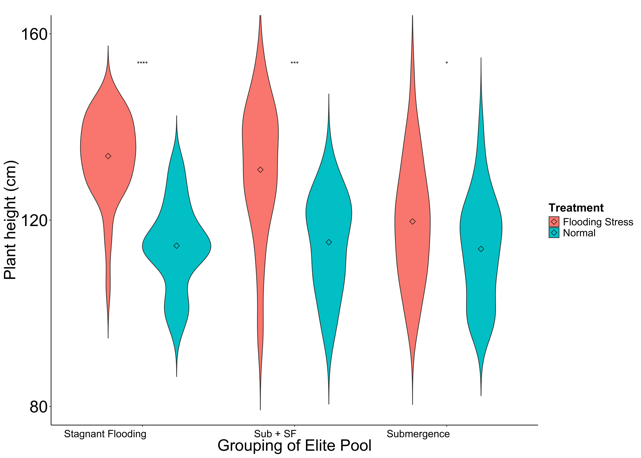


**Figure S3:** Distribution of days to maturity and plant height (cm) among the 89 elite genotypes under flooding and normal conditions. The X-axis shows the grouping of three different types of groups, and the y-axis shows the values for days to maturity and plant height. Different colors show the normal and flooding environments. In Figure, *ns* means non-significant (p>0.05), and *** (p<0.05), **** (p<0.01), and **** (*p<0.001) show significant differences.

**
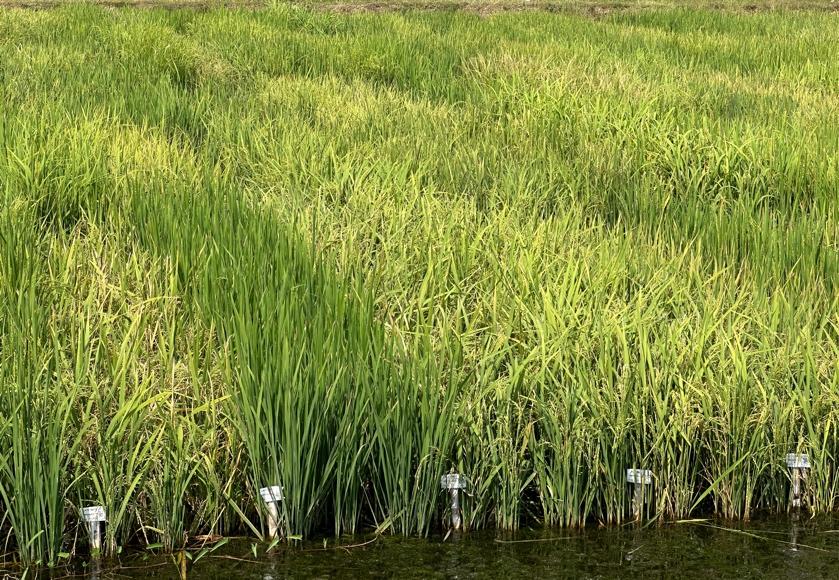

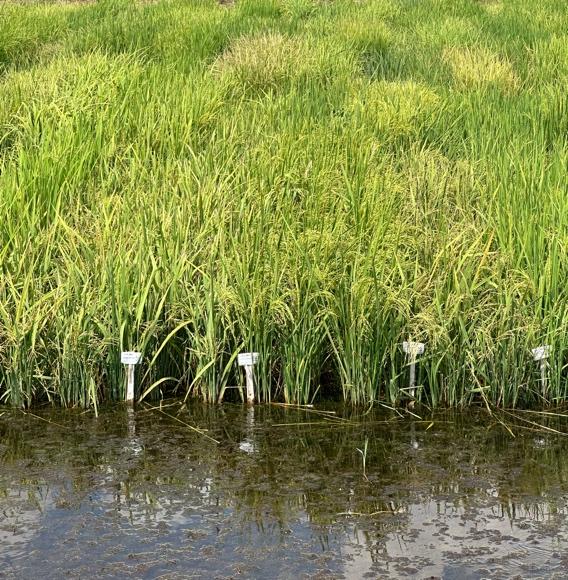
**

**Figure S4.** Visual representation of the tolerant stagnant flooding genotypes grown in the IRRI-HQ field during the 2023 dry season. Genotypes are tall, strong, erect, and compact, which are unique characteristic features of stagnant flooding tolerance.
