# Supplementary material for: Future flooding tolerant rice germplasm: Resilience afforded beyond Sub1A gene
Source: Plant Genome. 2025 May 8;18(2):e70040. doi: 10.1002/tpg2.70040 (PMC12060086; doi:10.1002/tpg2.70040)
Supplement: Supplementary file 3 — Supplementary Information [file TPG2-18-e70040-s002.docx]

**Supplementary Information**

**Future flooding tolerant rice germplasm: resilience afforded beyond *Sub1A* gene**

Mahender Anumalla^1,2^, Apurva Khanna^1^, Margaret Catolos^1^, Joie Ramos^1^, Ma Teresa Sta. Cruz^1^, Challa Venkateshwarlu^2^, Jaswanth Konijerla^2^, Sharat Kumar Pradhan^3^, Sushanta Kumar Dash^3^, Yater Das^4^, Dhiren Chowdhury^5^, Sanjay Kumar Chetia^6^, Janardan das^7^, Phuleswar Nath^7^, Girija Rani Merugumala^8^, Bidhan Roy^9,^ Navin Pradhan^10^, Monoranjan Jana^11^, Indrani Dana^11^, Suman Debnath^11^, Anirban Nath^12^, Suresh Prasad Singh^13,^ Khandakar Md Iftekharuddaula^14^, Sharmistha Ghosal^14^, Mohammad Ali^15^, Sakina Khanam^16^, Md Mizan Ul Islam^17^, Muhiuddin Faruquee^17^, Hosna Jannat Tonny^17^, Md Rokebul Hasan^18^, Anisar Rahman^14^, Jauhar Ali^1^, Pallavi Sinha^1,2^, Vikas Kumar Singh^1,12^_,_ Mohammad Rafiqul Islam^17^, Sankalp Bhosale^1^, Ajay Kohli^1^, Hans Bhardwaj^1^, and Waseem Hussain^1^*

**Submergence Screening Protocol with 21 Days Submergence Period**

In this submergence screening, rice is submerged under water for 21 days after 14 days of transplanting. The water level is raised rapidly for at least 1 meter for 21 days, wherein the temperature and turbidity of the floodwater are also monitored to avoid severe stress.

**
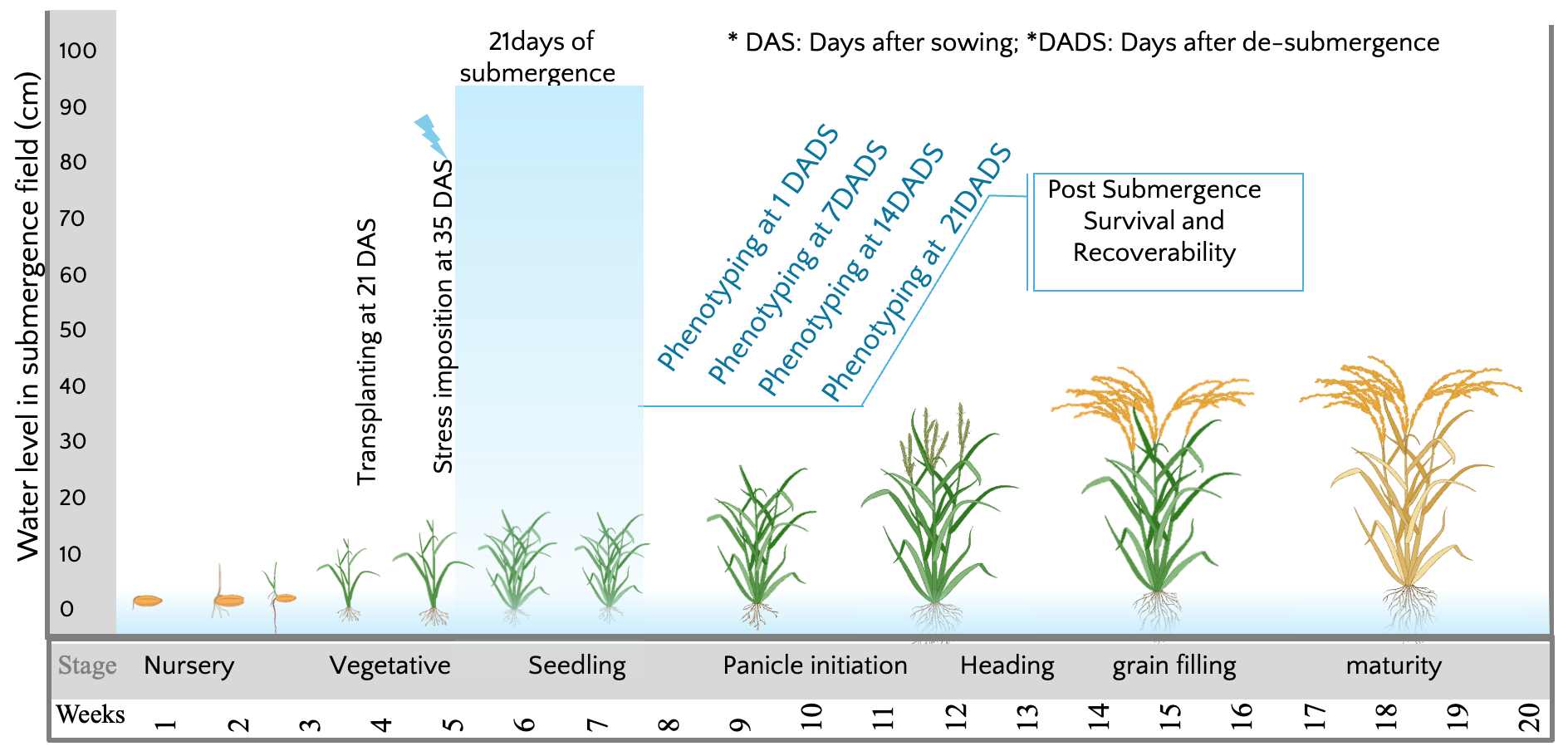
**

Diagram illustrating the 3-week submergence protocol. Rice seedlings are transplanted after 21 days. After 2 weeks of transplanting, submergence stress is applied for 21 days. Phenotyping and data collection occur at 1, 7, 14, and 21 days after de-submergence or water removal.

**General Guidelines**

**Land preparation for lowland fields:** Prepare the land thoroughly as recommended and level it before transplanting so that no low and high soil spots are observed after the final leveling.

**Experimental design:** Augmented RCBD or Alpha lattice with two replications (if the area is available)

**Checks:** The local and global checks to be replicated in each block

- Susceptible-high yielding lines: IR 42 , Ciherang, IR 64, Swarna
- Tolerant lines: FR13A, Ciherang-sub1, IR64-sub1, IRRI 119, Swarna Sub 1

**Sowing:** Raise healthy and robust seedlings as this impacts survival and recovery from submergence stress and may increase yield by up to 40%.

**Spacing between plants and rows:** 20 × 20 cm. Each plot should be maintained 3 or 5m2 .

Please follow the recommended dose of fertilizers as per the specific region.

**Cultural Management Practices**

1. The nursery should be raised as per the local management practices.
2. At 21-25 days old seedlings should be used for transplanting. One seedling per hill should be transplanted in a 5 m2 plot, and replanting is needed when there are missing hills in a plot. The gap-filling can be done in one week after transplanting to ensure 100% plant establishment. Susceptible checks (such as IR42, Swarna, etc.) are planted separately to check the damage due to Submergence.
3. The submergence stress is imposed 14 days after transplanting for 21 days, susceptible check is showing symptoms of deterioration (foul smell).
4. Before submerging the field tank, install at least 2 vertical water level gauges at strategic locations in the pond (side, middle, front, back) for monitoring the pond water depth.
5. Count the total number of plants (planted hills) per plot per entry before submerging the pond. Measure the height of five random plants in each entry.
6. Increase the water level of the pond to 1.0 m, 14 days after transplanting. If some leaves of the entries float 5-10 days after Submergence, increase the water level to 1.5 meters to prevent the escape of the tall entries.
7. Monitor the water quality (murkiness), pH, EC, and dissolved O2, if measuring devices are available. The depth of water is needed to maintain for the period of 21 days by adding the water regularly if the level of water has decreased.
8. Sample susceptible checks by pulling the pots planted to sensitive checks (Swarna) to observe the plant condition starting five days after Submergence (if the water is very murky), until such time that symptoms of deterioration (foul smell) are apparent. With severe submergence stress, the plants will be 70%-80% chlorotic, and the stems will be very soft. This condition is expected to show any day starting from 10 to 14 days of Submergence, depending on floodwater quality and environmental conditions.
9. Weeding will be done on the 7th and 9th day of de-submergence if needed.
10. Drain the field tank after the submergence stress treatment, that is, when the susceptible entry in the pots has deteriorated. Allow the field to remain without water for 3-4 days.
11. When the soil is already very dry for three days, and the plants start to recover, re-irrigate the field in about ten days from de-submergence, with an initial depth of 1-2 cm for 15-20 days. Increase the water level to 3-5 cm and maintain it until the hard dough stage.
12. Drain the field 1-2 weeks before harvesting to facilitate easier harvest.

**Fertilizer management before and after Submergence**

- During crop establishment, do not apply fertilizer after transplanting. Avoid excessive nitrogen applications. Soils in submerged areas usually are high in phosphorus, due to siltation.
- After de-submergence, apply fertilizer only after the plants have fully recovered, usually 7-14 days after water recession.
- Apply a half dose of nitrogen at 7-14 days after de-submergence and the remaining half dose of nitrogen at panicle initiation.

**Note: For induced or managed-submergence stress:**

- During crop establishment, do not apply basal fertilizer. Avoid excessive N application. Soils in submerged areas usually are high in P, due to siltation.
- After de-submergence, apply fertilizer ONLY after the plants have fully recovered, usually 7-14 days after water recession. With fertile soil, apply a half bag (25 kg) of urea (11.5 kg N per hectare). Have split N application, i.e., 12.5 kg urea applied at 7-14 days after de-submergence and the remaining 12.5 kg urea at panicle initiation stage.
- When in need of more nutrients, depending on the status of the plants after de-submergence, use any available information on soil nutrient status (soil analysis, recommended rate for the area, MOET), or fertilizer responses to decide on which nutrient and how much is needed. As a guide, apply about 30-50 kg of N2, 20-30 kg P2O5, 20-30 kg of K2O per ha.

**Non-stress trial:**

A non-stress trial should also be laid out along with the stressed trial (as described above) for comparing the performance of genotypes under stressed and non-stressed conditions. The cultural management practices remain the same. However, stress should not be imposed.

**Data needed to be recorded:**

| **Trait** | **SES Scores** | **Observation** |
| --- | --- | --- |
| Phenotypic acceptability (PAcp) | 1 | Excellent |
|  | 3 | Good |
|  | 5 | Fair |
|  | 7 | Poor |
|  | 9 | Not Acceptable |
| Days to 50% flowering (FLW50Days) | The number of days from seeding to 50 % flowering. 50% of the primary tillers of the whole population (in a plot) are flowering | |
| Plant height (Ht) | Average of five samplings measured in centimeters from the soil surface to the tip of the tallest panicle (awns excluded) and can be measured when 80% of tillers are mature | |
| Number of hills harvested (NHs) | the actual number of hills harvested are recorded during harvest. | |
| Grain Moisture Content | Record moisture content after drying at 50^o^C | |
| Plot Yield | The total grain yield per plot is to be measured and expressed in grams. | |
| Survival percentage | count the number of survivors after 21 days of de-submergence  $\boldsymbol{\%Sur=}\left( \frac{\boldsymbol{Actual no. of hills with surviving plants}}{\boldsymbol{Total number of hills before submergence}} \right)\boldsymbol{\times100\%}$ | |

Reference: Standard Evaluation System for Rice (SES), International Rice Research Institute (IRRI),5th Ed. June 2013

**Record all observed insects and pests damage observed during the duration of the trials.**

**Harvesting, Drying, and Threshing:**

- Harvest the whole plot except the front and back rows of the plot to avoid border effects on the yield. Please be reminded to record the number of hills harvested for each plot and harvest date.
- Harvested samples should be appropriately labeled using the printed harvested tags, which should at least include the trial number and plot number. The harvest tag should match the field tag corresponding to the plot, and both tags should be placed inside the net bag during harvest.
- Harvested samples should be placed inside the oven for drying at 50C for 2-3 days and then threshed using the appropriate thresher machine.
- Threshed samples should then be cleaned using a seed blower. Clean seeds should be placed inside the properly labeled paper bag. The printed harvest tag should also be placed inside the paper bag.
